# Type 3 ILCs in Lung Disease

**DOI:** 10.3389/fimmu.2019.00092

**Published:** 2019-01-29

**Authors:** Amanda Ardain, James Zachary Porterfield, Henrik N. Kløverpris, Alasdair Leslie

**Affiliations:** ^1^Africa Health Research Institute, Durban, South Africa; ^2^College of Health Sciences, University of KwaZulu-Natal, Durban, South Africa; ^3^Yale School of Public Health, Yale University, New Haven, CT, United States; ^4^Department of Immunology and Microbiology, University of Copenhagen, Copenhagen, Denmark; ^5^Department of Infection and Immunity, University College London, London, United Kingdom

**Keywords:** ILC3, lung disease, pnemonia, tuberulosis, COPD, airway hypersensitivity, pulmonary fibrosis

## Abstract

The lungs represent a complex immune setting, balancing external environmental signals with a poised immune response that must protect from infection, mediate tissue repair, and maintain lung function. Innate lymphoid cells (ILCs) play a central role in tissue repair and homeostasis, and mediate protective immunity in a variety of mucosal tissues, including the lung. All three ILC subsets are present in the airways of both mice and humans; and ILC2s shown to have pivotal roles in asthma, airway hyper-responsiveness, and parasitic worm infection. The involvement of ILC3s in respiratory diseases is less well-defined, but they are known to be critical in homeostasis, infection and inflammation at other mucosal barriers, such as the gut. Moreover, they are important players in the IL17/IL22 axis, which is key to lung health. In this review, we discuss the emerging role of ILC3s in the context of infectious and inflammatory lung diseases, with a focus on data from human subjects.

## Introduction

The lungs are complex organs, the proper functioning of which is essential for good health, but whose exposure to the external environment renders them susceptible to infections and environmental toxins. The average person will breathe 11,500 L of air into their lungs in a given day. This air is full of small particulate matter that includes more than just the bacteria and viruses most typically associated with a lung immune response, and also includes dust, dirt, smoke, pollen, aerosols, and liquid droplets. The lung immune environment must therefore strike a delicate balance between dealing with this constant assault, whilst preventing immune mediated damage. This results in an intricate immune setting that is still not completely understood (1), but which growing evidence suggests includes an important role for innate lymphoid cells (ILCs).

**Figure 1:**
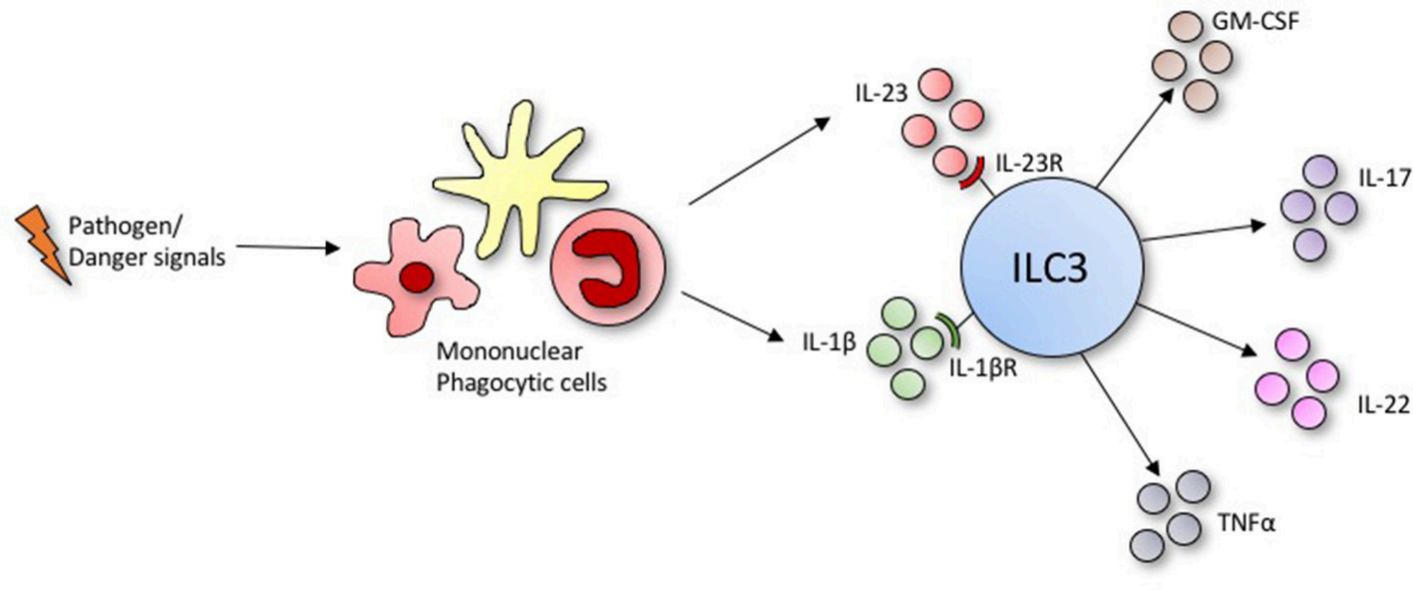
Under conditions of stress or infection, ILC3s produce an array of effector cytokines in response to activating ligands secreted by phagocytic mononuclear cells.

Innate lymphoid cells (ILCs) are a family of innate immune cells that lack specific antigen receptors but produce a broad range of effector cytokines and have diverse roles at barrier surfaces. ILCs mediate protective immunity from pathogens and promote tissue repair and homeostasis following infection, but may also play a role in pathogenesis when their functions become dysregulated (2–4). Broadly, ILCs are classified into three subsets based on surface markers, cytokine production, and transcription factor requirements (5). ILC1s are classically associated with the immune response to viral infection and tumor cells, while ILC2s are often compared to CD4^+^ Th2 cells and mediate the response to helminth infections and allergies. ILC3s are the most heterogeneous subset of ILCs, defined by expression of the Retinoid-related orphan receptor γt (RORγt), and produce IL-17, IL-22, granulocyte-macrophage colony stimulating factor (GM-CSF) and/or tumor necrosis factor α (TNFα) in response to IL-23, IL1-β and aryl hydrocarbon receptor (AHR) ligands (2, 6) (Figure 1). ILC3s can be broadly categorized into two lineages: the lymphoid tissue inducer cells (LTi cells) including fetal and adult-equivalent LTi-like cells (7), that have established roles in lymphoid organogenesis and secondary lymphoid tissue development, respectively; and non-LTi ILC3s which may or may not express natural cytotoxicity receptors Nkp44 and/or Nkp46 (8). ILC3s are widely distributed throughout the body and are constitutively present at mucosal barrier sites such as the lung, liver, gut, spleen, skin and secondary lymphoid tissues (9), where they play crucial roles in immunity and tissue homeostasis through the production of cytokines and interaction with the adaptive immune system (10).

**Figure 2:**
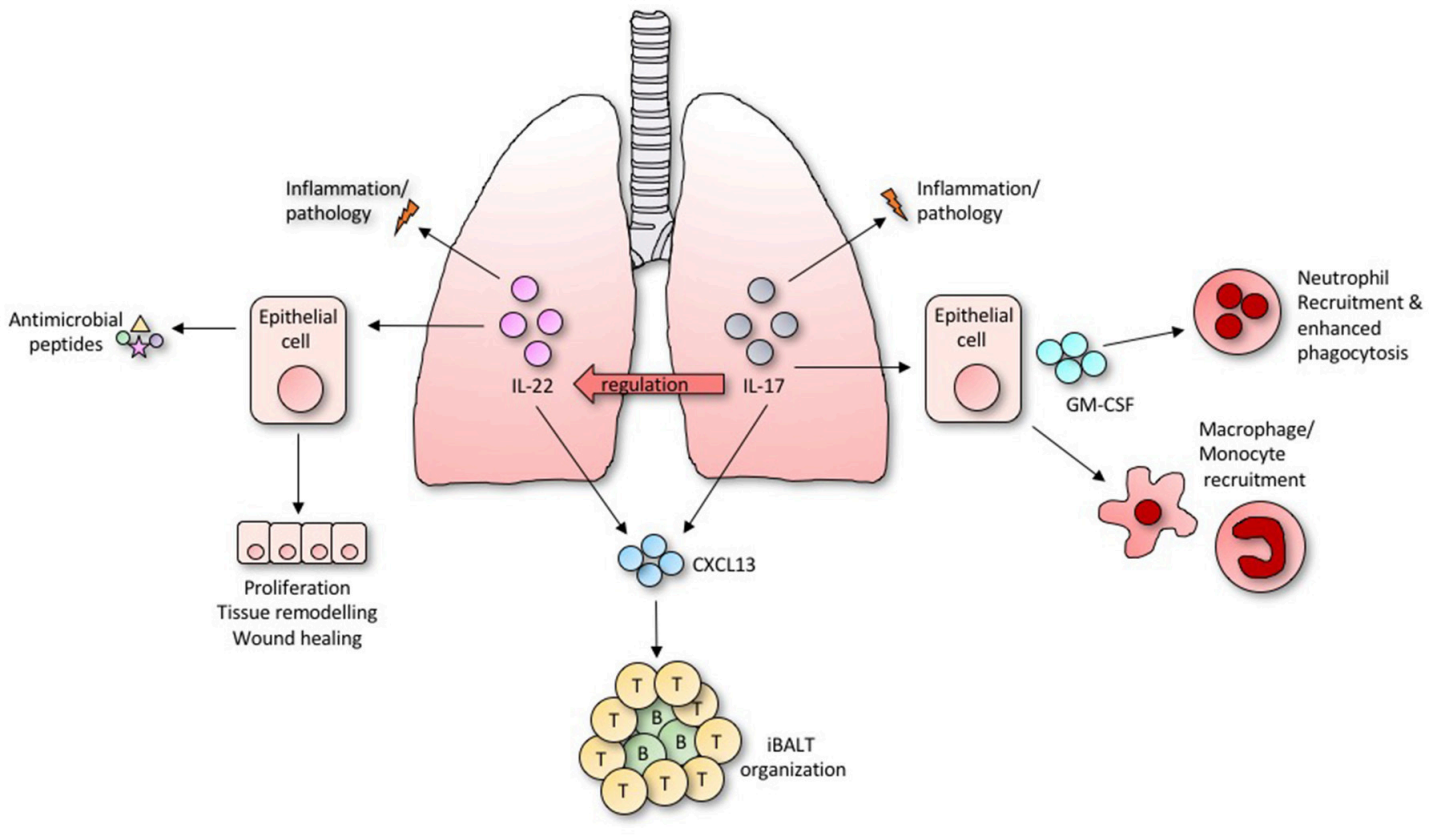
Diverse and overlapping roles of IL-22 and IL-17 within the lung.

While all three ILC subsets have been identified in the airways, the majority of studies to date have focused on ILC2s, highlighting their importance in tissue repair, protection from helminth infections, and involvement in multiple allergic diseases (3, 11, 12). However, ILC3s are in fact the most prevalent group in human lung tissues (13), and their rapid secretion of IL-17A and IL-22 has prompted investigations into their involvement in inflammatory and infectious diseases in the lung, as the IL-17/IL-22 axis is so important to lung health (14) (Overview in Figure 2). Moreover, GM-CSF also plays an important role in allergic airway disease, antimicrobial pulmonary host defense function, and is essential for surfactant homeostasis (15). Although the importance of ILCs as a source of GM-CSF in the lung remains untested, ILC3s in the gut are known to orchestrate inflammation through its activity (6).

## Infectious Diseases

The constitutive presence of ILC3s at mucosal barriers, including the lung, and their rapid production of cytokines allows them to efficiently orchestrate inflammation and antimicrobial peptide production, providing essential protection at these mucosal sites (4, 16, 17). Here we discuss several important human lung infections in which ILC3s may play an important role.

### Viral Infections

The influenza viruses are some of the most important human respiratory pathogens, causing substantial seasonal, and pandemic morbidity and mortality (18). Following infection, the innate immune system provides the first line of defense, with various innate cell types playing important roles in killing infected cells and/or limiting viral replication. However, an excessive innate immune response correlates with poor outcomes and can lead to pathology (19, 20), and lung regeneration following viral-induced injury is vital (18). ILC2s have been best studied in relation to influenza, due to their ability to mediate tissue repair following Influenza infection via the production of amphiregulin (3). However, both IL-22 and IL-17 are implicated in the immune response to pulmonary viral infection, suggesting a potential role for ILC3s, particularly prior to the onset of the adaptive immune response or in primary infection. Mice infected with Influenza A/PR/8, for example, produced IL-17A as early as 2 days post-infection, resulting in lung injury associated with excessive neutrophil recruitment (21), and IL-17 deficiency or treatment with anti-IL-17 antibodies is able to ameliorate IL-17 associated lung injury in infected mice (22). However, as with many things in immunology, this is a double edged sword, as IL-17 also plays an important protective role in preventing secondary bacterial infections. Two separate studies have shown in co-infection experiments with influenza and *Staphylococcus aureus*, that the induction of type I interferon from the virus increased susceptibility to secondary bacterial pneumonia by directly inhibiting IL-17 production (23, 24). Irrespective, IL-17 is likely to be relevant in human influenza infection, as elevated levels of IL-17 and IL-17 associated cytokines are detected in serum from humans infected with swine-origin Influenza virus (22). As antigen-specific CD4^+^ T cells do not appear in the lung earlier than 4 days post-infection (25), innate sources of IL-17 are likely to play an important role. γδ T cells have been implicated as being the early source of IL-17 (21, 26) however, although not directly linked as yet, it is likely that ILC3s represent an additional source of IL-17 during the first week of an influenza infection, particularly in primary infections.

IL-22, also has critical functions in host defense and in maintaining epithelial integrity during influenza infection (10, 27). In mice infected with a sub-lethal dose of H3N2 Influenza A, ILCs had enhanced expression of *IL-22* transcripts as early as 2 days post-infection, and mice able to produce IL-22 had reduced lung injury and better protection from post-influenza *Streptococcus pneumoniae* superinfection, in comparison to IL-22-deficient animals (28). In another study, IL-22 knock out mice were found to have exacerbated lung injury compared with wild-type mice, correlating with decreased lung function 21 days post-infection with a PR8/34 H1N1 virus (29). Similarly, Kumar et al. (30) found that IL-22^−/−^ mice infected with a PR8 Influenza virus had severely impaired epithelial regeneration and continuous loss of body weight, which was restored by adoptive transfer of IL-22 sufficient, “ILC3-like” CD3^−^NCR1^+^NK1.1^+^ cells. Guo and Topham (31), also identified a CD3-NCR1+NK1.1+ subset as the predominant producers of IL-22, although the protective role of IL-22 was less clear in this instance. Subsequently, transcription factor staining and transcriptomics of mucosal barrier surfaces have demonstrated that ILC3s are a major source of IL-22 (32).

### Bacterial Pneumonia

Pneumonia remains one of the largest infectious causes of death globally, accounting for the deaths of 16% of children under 5 years old (33).

The severity of pneumonia depends on the interplay between the ability of the host to control infection and the extent to which the stress of microbial challenge can be tolerated (34). IL-17 is important in the control of several key bacterial causes of pneumonia in humans, including *Klebsiella pneumoniae, Streptococcus pneumoniae*, and *Pseudomonas aeruginosa* (23, 35–37). Humans with inherited complete autosomal recessive IL-17RA deficiency, for example, have increased susceptibility to pneumonia (38). In mechanistic studies, IL-17R knockout mice were found to be particularly susceptible to *K. pneumonia*, demonstrating 100% mortality after just 48 h in comparison to 40% mortality amongst controls (39). This was attributed to a considerable delay in alveolar neutrophil recruitment via IL-17. Similarly, IL-17R deficient mice, or mice with depleted neutrophil populations had significantly diminished protection from pneumococcal infection in the lung, and IL-17 is known to enhance the phagocytic activity of neutrophils *in vitro* (40). These observations are supported by recent work showing that IL-17 signaling in the lung epithelium plays a critical role in establishing the chemokine gradients that are essential for mucosal immunity against pulmonary bacterial pathogens. Here, mice that lacked IL-17R expression specifically on lung epithelia lost CXCR5 signaling and failed to recruit sufficient neutrophils to clear *K. pneumoniae* (36). The protective effect of IL-17 is not only limited to the activity neutrophils. Clearance of primary infection with *S. pneumoniae*, for example, requires the recruitment of monocyte/macrophages, and this protective response is blocked by the depletion of IL-17 (41). Again, while Th17 and γδ T-cells are important sources of IL-17 in the recall response to bacterial infection (37, 42), in primary infections, other sources, including ILC3s are likely to be crucial. This is highlighted by the recent work of Xiong et al. (43) demonstrating the importance of ILCs in primary infection with *K. pneumoniae*. This study described a positive feedback loop in which TNF-producing monocytes recruited to lungs drove an increase in IL-17-secreting ILCs, which in turn enhanced monocyte-mediated bacterial uptake and killing. Importantly, bacterial clearance was similar in T-cell deficient and WT mice, but depletion of ILC3s led to increased bacterial burden and mortality (43). In experimental infection with *P. aeruginosa*, for which IL-17 is also essential, 90% of early IL-17 production appears to come from ILC3 rather than CD3+ lineages, including γδ T-cells (35).

As with viral infection, IL-22 also plays key roles in host response to bacterial pneumonia. In mice, IL-22 is detected in lungs as early as 6 h post-infection, where it increases lung epithelial cell proliferation and significantly augments killing of *K. pneumoniae in vitro* by upregulating the expression of lipocalin-2 (44). The early expression of IL-22 implicates an innate source. Following on from this Van Maele et al. (32), using *Rag*^−/−^ and *Rag*^−/−^*/Il2rg*^−/−^ mice, demonstrated IL-22 production by CCR6+ ILC3s during *S. pneumoniae* infection. Interestingly, this ILC3 response could be boosted by exogenous administration of the TLR ligand flagellin, and was sufficient to control and protect from an otherwise lethal infection (32). This raises the intriguing possibility that modulation of ILC3s may represent a novel treatment modality for some lung infections. The potential interplay between IL-22 and IL-17 in bacterial pneumonia is highlighted by recent work showing that, whilst IL-17 driven neutrophil recruitment maybe required for bacterial clearance, infection with *P. aeruginosa* in the absence of IL-22 is associated with pathogenesis and increased susceptibility driven by an excessive neutrophil response (45). Finally, IL-22 production by ILC3s was recently found to be vital in neonates, being the primary source of this cytokine in both mice and humans. In this study, recruitment of IL-22-producing ILC3s to the lung, driven by commensal microbes in the gut, was required for protection from *S. pneumoniae* infection (46). This unexpected link between the gut microbiome and ILC3-mediated immunity in the lung again raises the possibility of manipulating ILC3s to improve lung health. It also demonstrates, as with primary infection, that the importance of ILC3 produced cytokines may vary depending on the context.

### Tuberculosis

Tuberculosis (TB), despite typically being a fully treatable disease, continues to contribute to more deaths worldwide each year than any other infectious disease (47), pulmonary or otherwise. While the immune response to TB is complex and not fully understood, human and mouse models have progressively elucidated roles for many different immune cells including alveolar macrophages, epithelial cells, and CD4+ T cells (48, 49). Studies of ILCs in this mix are extremely limited to date. However, ILCs are activated and accumulate in the lungs and lymph nodes of mice following intranasal vaccination with *Mycobacterium bovis* Bacillus Calmette-Guérin (BCG) (50). Moreover, their roles in lung tissue repair, hepatic granuloma formation (3, 51), and pro-inflammatory cytokine production support their involvement. Indeed, prior to the description of ILCs, Feng et al. (52) found that *Rag*^−/−^γ*c*^−/−^ mice, which lack T-cells and all ILC subsets, had significantly poorer survival than T-cell deficient *Rag2*^−/−^ mice or WT controls, reflected in a more than 2-fold increase in lung bacterial burden. Consistent with this, whilst Th1-associated molecules, such as TNFα and IFNγ are crucial to mycobacterial resistance (53–55), recent studies have extended this to include IL-17 and IL-22. BCG-infected mice, for example, fail to develop mature granulomas in the absence of IL-17 (56), and both IL-17 and IL-22 protect mice from the hypervirulent strain *Mtb* HN878 (57, 58). Using cells sorted from human lung tissue explants, ILC3s have recently been show to upregulate GM-CSF and IL-22 transcripts when stimulated with *Mtb ex vivo* (48), and IL-22-producing NK cells, now recognized as ILC3s, inhibit *Mtb* growth by enhanced phagolysosomal fusion (59). Additionally, humans with inherited bi-allelic RORC mutations, which lead to compromised IL-17A/F mediated immunity, have markedly increased susceptibility to mycobacterial disease (60).

One potential mechanism by which ILCs may contribute to the immune response to TB infection is through the formation of inducible bronchus-associate lymphoid tissue (iBALT). These are tertiary lymphoid structures that develop following pulmonary inflammation, at least in part through the IL-17/22 axis, and which appear to orchestrate protective immune responses (57, 61–63). Several independent studies have highlighted the importance of iBALT formation in the immunopathology of TB. Mice deficient in IL-17 had reduced B cell follicle formations during early stages of *Mtb* challenge, while IL-22-deficient mice had impaired follicle formation at an intermediate stage, although neither of these deficiencies impacted the mouse's ability to control the infection. A lack of IL-23, which induces cytokine production by ILC3s, led to both a sustained follicle deficiency and increased bacterial growth (64). Separately, IL-17, and not IFNγ, was found to be required for mucosal vaccine-induced immunity against *Mtb*, and was associated with the induction of organized iBALT structures in the lung (65). Thus, early and/or sustained production of IL17/IL22 by ILC3s may play and important role in iBALT formation during *Mtb* infection. It is important to note, however, that iBALT formation can be observed in neonatal RORγt KO mice (66), showing that ILC3s are not absolutely required for this process.

## Autoimmune Disease

ILC3s have been associated with several autoimmune diseases, including inflammatory bowel disease, multiple sclerosis, psoriatic arthritis, and ankylosing spondylitis, either by direct evidence or by involvement of the IL17/IL22 axis (67–70). ILCs are also implicated in numerous autoimmune diseases which can have lung associated pathology, including systemic sclerosis, systemic lupus erythromatosus, and the ANCA-associated vasculitides, however, the relationship is most clearly worked out in asthma, COPD, and pulmonary fibrosis.

### Asthma and Airway Hyper-Reactivity

Asthma is a chronic respiratory disease characterized by airflow obstruction, excessive inflammation and airway remodeling (71). IL-17 appears to be an important cytokine driving this this condition. IL-17 expressing cells, for example, are elevated in both sputum and Bronchoalveolar lavage fluid (BALF) from asthmatic patients, (72) and more recently, IL-17+ ILC3s in particular were found to be increased in BALF from human patients with severe asthma (73). Consistent with this, ILC3 gene signatures are enriched in total RNA from patients with adult onset asthma (74). In mice, ILC3s facilitate obesity-linked asthma via IL-17 production. Here, in both wild-type mice fed a high fat diet, and mice that are obese due to a mutation in the leptin gene, airway hyperresponsiveness was found to mediated by IL-17+ ILC3s (73). Importantly, this was observed in T-cell deficient mice and was induced by adoptive transfer of ILC3s into mice lacking all lymphoid cells. Subsequently, IL-17-producing ILC3s were similarly implicated in allergic asthma. Here, IL-17+ ILC3s and ILC2s were increased in mice fed a high fat diet following house dust mite challenge, and this was ameliorated by antibody depletion of ILCs using anti-CD90 (75).

IL-22 expression is associated with increased eosinophil recruitment to lungs and airway hyper-responsiveness, and can be detected in mice with ovalbumin induced allergic asthma (76), and in serum from human patients (77). The major source of IL-22 in mouse models is yet to be confirmed, as one study identified the origin to be CD4+ T cells (78), while another study found it was predominantly produced by lineage negative, CD90^+^, Sca-1^+^ ILCs (79). However, in both studies, as well as others, neutralization of IL-22 led to increased eosinophil infiltration and allergic inflammation, while treatment with recombinant IL-22 ameliorated this response (76–79). Here, the implication that IL-17 is detrimental and IL-22 protective in these situations is likely to be a simplistic one due to the cross talk between these cytokines. Besnard et al. (77), for example found that IL-22 played a protective role in the initial stages following antigen challenge, but exacerbated inflammation in the later stages.

### COPD

Chronic obstructive pulmonary disease (COPD) is largely caused by smoking, and is associated with an aberrant inflammatory lung environment (80), characterized by lung destruction and increased neutrophilic infiltration (81). Consistent with their roles in tissue immunity and homeostasis, recent studies have identified roles for ILCs in COPD, particularly ILC1s (82). However, ILC3s may also be involved, as IL-17 is recognized as a key driver of neutrophilic inflammation in COPD (83). In mice, cigarette smoke exposure leads to an increase of all ILC subsets in BALF, particularly IFNγ+ and IL-17+ ILCs (84). In line with this, human patients with COPD have higher sputum IL-17 than non-smoking controls (85), and IL-17-, IL-22-, and IL-23-expressing cells are increased in bronchial biopsies from COPD patients (81). While direct mechanistic evidence linking ILC3s to COPD is limited, one human study showed that a subset of ILC3s was notably increased in lung tissue from COPD patients, and an increase in IL-17 and IL-22 production by ILC3s (13). In separate work, gene set enrichment analysis revealed LTi ILC3s and NK genes are enriched in lymphocytes sorted from human lungs tissues of patients with severe COPD (86).

### Pulmonary Fibrosis

Pulmonary fibrosis occurs as a result of damage and scarring of the lung parenchyma that can have numerous causes including, environmental toxins, medication side effects, radiation therapy, and autoimmune conditions. In fibrotic disease, chronic inflammation and persistent extracellular matrix deposition can lead to remodeling and progressive tissue destruction (87). This remodeling and extracellular matrix deposition is largely driven by connective tissue cells, such as fibroblasts, whose functions are directly affected by pro-fibrotic cytokines produced by adaptive and innate immune cells (88). The types of inflammatory processes facilitated by ILCs, and their cytokine expression suggests they are substantial contributors to fibrosis (87). While all three ILC subsets secrete fibroblast activating cytokines, ILC3s have capacity to regulate synovial fibroblasts and connective lung tissue cells via IL-17 (89, 90), and elevated levels of IL-17 have been observed in patients with cystic and pulmonary fibrosis (91, 92). In a mouse model of fibrosis, IL-17 was produced early on (between days 2 and 7), and associated with neutrophil influx into the lungs. Mice deficient in IL-17 had lower neutrophil frequencies and better disease outcomes (92). It seems that timing is important, however, as treatment with IL-17 has no effect on collagen deposition during chronic disease (93).

IL-22 is also involved in fibrosis, again in a largely protective role. In a mouse model of hypersensitivity pneumonia, for example, blockade of IL-22 signaling caused accelerated lung fibrosis and enhanced collagen deposition in the lung, which was inhibited by the administration of recombinant IL-22 (94). Similarly, bleomycin treatment in mice, which induces fibrosis, significantly reduced IL-22 in their lungs, and human epithelial cell lines treated with IL-22 had a slower progression to fibrosis (95). However, again this protective view of IL-22 is probably simplistic as one study found that bleomycin induced IL-22 production was only protective in IL-17 deficient mice (96). Conversely, in the presence of IL-17, IL-22 was proinflammatory and promoted airway pathology, which was blocked by the administration of anti-IL-22 antibody. Clearly then, the involvement of IL-17 and IL-22 in the lung should not be considered separately. As important innate sources of both cytokines in the lung, it appears likely that ILC3s would have a role in pulmonary fibrosis and they produce represent promising new targets for molecular therapies (92, 94).

## Concluding Remarks

As we have highlighted here, there are many studies showing the importance of ILC3 cytokines in lung immunity. In many cases the early source of cytokines such as IL-17 and IL-22 has been assumed to be γδ T cells, as antigen-specific T cells only accumulate after several days (25). However, we now know that ILC3s, though fewer in number, are prolific producers of IL-17 and/or IL-22, as well as other important lung cytokines such as GM-CSF, and thus have the capacity to drive inflammation or repair in a number of infectious and autoimmune diseases (97–100). This is certainly supported by the relatively few studies to date that have directly sought to investigate their importance as a source of these cytokines. Clearly much remains to be done, but the possibility that lung ILC3s may be manipulated to influence lung health is intriguing and should encourage these further studies.

## Author Contributions

AA and JP: Literature research and writing; HK and AL: Literature research and editing.

### Conflict of Interest Statement

The authors declare that the research was conducted in the absence of any commercial or financial relationships that could be construed as a potential conflict of interest.
